# Natural compounds in ovarian cancer: mechanistic insights and therapeutic potential

**DOI:** 10.3389/fphar.2025.1687805

**Published:** 2026-01-22

**Authors:** Barathan Muttiah, Nur Atiqah Haizum Abdullah

**Affiliations:** 1 Department of Medical Microbiology and Immunology, Faculty of Medicine, Universiti Kebangsaan Malaysia, Kuala Lumpur, Malaysia; 2 Department of Tissue Engineering and Regenerative Medicine, Faculty of Medicine, Universiti Kebangsaan Malaysia, Kuala Lumpur, Malaysia

**Keywords:** natural compounds, apoptosis, nanodelivery systems, chemoresistance, ovarian cancer

## Abstract

Ovarian cancer is one of the most lethal gynecologic malignancies due to its late diagnosis, high recurrence rate, and chemoresistance. Recently, increasing evidence has emphasized the therapeutic potentials of natural compounds as multi-targeted agents in modulating key oncogenic pathways and improving standard therapies. This review critically examines the anticancer properties of various NCs, including quercetin, curcumin, resveratrol, EGCG, berberine, ellagic acid, withaferin A, celastrol, and others, against OC. These compounds display broad-spectrum activities: inhibition of cell proliferation, induction of apoptosis, modulation of oxidative stress, suppression of angiogenesis and metastasis, and reversal of chemoresistance. At the mechanistic level, NCs modulate several signaling pathways, such as PI3K/AKT/mTOR, NF-κB, MAPK, and Wnt/β-catenin pathways; and influence epigenetics and microRNA-mediated mechanisms. In contrast to compelling preclinical evidence, clinical translation remains limited due to poor bioavailability, the absence of OC-specific clinical trials, and regulatory constraints. The focus of future research should be on advanced drug delivery systems, omics-guided precision medicine, and sustainable sourcing strategies to overcome these translational barriers. The integration of NCs into combination and personalized regimens has promise for the improvement of therapeutic outcomes and overcoming chemoresistance in ovarian cancer.

## 1 Ovarian cancer

Ovarian cancer (OC) is a serious and potentially life-threatening disease that affects women globally. It is the eighth most common cancer in women and the fifth leading cause of cancer-related deaths among women (Zhang et al., 2022). The global rise in OC presents a significant public health challenge, with new cases expected to increase by 42% by 2040, from 313,959 in 2020 to 445,721. Similarly, mortality rates are projected to rise by 51%, from 207,252 deaths in 2020 to 313,617 by 2040 (Cabasag et al., 2022). There are several risk factors associated with OC, including family history, genetic mutations (such as BRCA1 and BRCA2), age, obesity, hormone replacement therapy, and a history of endometriosis (Ali et al., 2023). The high mortality rate associated with OC is largely due to delayed diagnosis, as the disease is often asymptomatic until it reaches advanced stages characterized by ascites and metastasis (Giro et al., 2025). This late-stage diagnosis is linked to a poor prognosis and a 5-year survival rate of less than 30%. Diagnosis of OC typically involves a physical exam, imaging tests (such as ultrasound or CT scan), blood tests (including CA-125), and a biopsy to confirm the presence of cancer cells, however the current screening methods, including transvaginal ultrasound and CA-125 testing, have limited predictive value, underscoring the challenges in achieving early detection and effective intervention (Hong and Ding, 2025).

The treatment of OC involves a multidisciplinary approach, starting with surgery to remove the ovaries, fallopian tubes, uterus, and sometimes nearby lymph nodes or other affected tissues (Engberse et al., 2021). Chemotherapy often follows surgery or is used to shrink tumors beforehand, with common drugs including platinum-based agents like carboplatin and taxanes such as paclitaxel (Chandra et al., 2019). Targeted therapies, such as PARP inhibitors for BRCA-mutated cancers and bevacizumab to inhibit blood vessel growth, are increasingly employed (Wang et al., 2025). Immunotherapy is an emerging option that enhances the immune system’s ability to fight cancer, though its use in OC is still being explored (Ghemrawi et al., 2024). Radiation therapy, though less common, may be utilized to shrink tumors or manage symptoms in specific cases (Durno and Powell, 2022). Hormone therapy is sometimes used to slow the growth of hormone-sensitive ovarian cancer cells, particularly in cases like low-grade serous carcinoma (Ottenbourgs et al., 2024). While several reviews have highlighted the anticancer activity of natural products, the present paper differs in combining mechanistic insight with discussion of leading-edge delivery systems such as nanoparticles, extracellular vesicles (EV) and issues of translation. By weighing molecular mechanisms against clinical shortfalls, it aims to provide a comprehensive guide to promoting the clinical application of natural products in the treatment of OC.

## 2 Traditional medicine as alternative therapy

Traditional medicine in India, China, and Egypt has long utilized plant treatments, and consequently they possess a very extensive pharmacological database that continues to guide modern-day oncology (Rizvi et al., 2022). The medicinal use of plants not only led to treatment but also foreordained the identification of active anticancer agents (Khan et al., 2019). For example, paclitaxel (Taxol®), which is derived from the bark of Pacific yew (Taxus brevifolia), revolutionized ovarian cancer treatment after purification by semi-synthetic preparation and formulation methods (Sati et al., 2024). Likewise, camptothecin, which was originally purified from Camptotheca acuminata in Chinese traditional medicine, resulted in the creation of the clinically used analogs topotecan and irinotecan, which are now used in ovarian and other solid malignancies (Gong et al., 2025). These examples show how ancient therapies led to modern chemotherapeutics, bridging ancient knowledge with the drug discovery pipelines of today (Teppone, 2019). They also provide valuable lessons for the natural compound research of today: the need for sustainable availability of resources, chemical optimization towards greater bioavailability, and rigorous clinical validation to turn back-of-basics knowledge in history into therapeutic reality (Falzone et al., 2018).

## 3 Mechanistic insights into the anticancer effects of NCs in OC

NCs have indeed shown potent anticancer activities against ovarian tumor cells and their TME (Moody et al., 2020). Due to their structural diversity, multiple signalling pathways are targeted simultaneously with greater specificity and lower toxicity when compared to conventional chemotherapeutic agents (Andreani et al., 2024; Ahmed et al., 2022).

Induction of programmed cell death is a principal mechanism. NCs induce intrinsic mitochondrial apoptosis via release of cytochrome c and activation of caspases and extrinsic receptor-mediated apoptosis through TNF receptor signalling, thereby overcoming the resistance to apoptosis in recurrent OC. These include procyanidins, zeylenone, sanguiin H-6, and tanshinones (Islam et al., 2023; Molto Valiente and Amir, 2023).

Furthermore, NCs exert cell cycle arrest, either at the G0/G1 or G2/M phase, while also inhibiting the genes for proliferation and suppressing oncogenic signalling pathways such as PI3K/Akt, MAPK, JAK/STAT3, NF-κB, mTOR, and Wnt/β-catenin. For example, curcumin nanoparticles inhibit PI3K/Akt and STAT3 pathways; quercetin and berberine hinder EMT, thereby reducing metastasis (Gao et al., 2021; Wu et al., 2021).

Another important mechanism is the regulation of autophagy (Xie et al., 2021). Resveratrol, withaferin A, and tanshinones modulate Beclin-1 and LC3 expression, shifting the balance toward autophagic cell death, often synergizing with apoptosis to eliminate chemoresistant cells (Wu S. et al., 2023). NCs also enhance chemosensitivity via blocking efflux pumps, for instance, P-glycoprotein; impairing DNA repair mechanisms; and reactivating proapoptotic pathways. Withaferin A enhances doxorubicin efficacy, while resveratrol and sulforaphane reverse cisplatin resistance (Fong et al., 2012).

Beyond direct cytotoxicity, NCs remodel the TME via inhibition of angiogenesis through suppression of VEGF and HIF-1α, reduction of inflammation via inhibition of COX-2 and IL-6, and modulation of immune cell recruitment to favour antitumor immunity (Lu et al., 2025a). Some flavonoids and phenolics selectively induce oxidative stress in neoplastic cells while sparing normal tissue (Silva-Pinto et al., 2025; Tavsan and Kayali, 2019).

**FIGURE 1:**
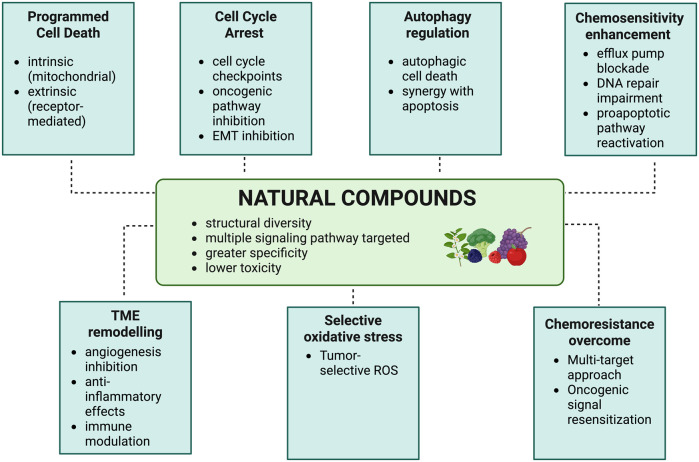
The seven mechanistic insights into the anticancer effects of natural compounds (NCs) in ovarian cancer (OC).

Overall, the low-toxicity multitargeting capability underlies the power of NCs in overcoming OC chemoresistance: by suppressing drug efflux, targeting cancer stem cells, inducing alternative death pathways like ferroptosis, and re-sensitizing tumors through inhibition of hyperactive oncogenic signaling. This multimodal activity provides a comprehensive therapeutic approach against both tumor progression and drug resistance in OC (Debnath and Kundu, 2025; Cui et al., 2025). Schematic illustration summarizing seven key mechanistic insights underlying the anticancer effects of natural compounds (NCs) in ovarian cancer (OC) was shown in Figure 1.

### 3.1 Ferroptosis as an emerging anticancer mechanism

**FIGURE 2:**
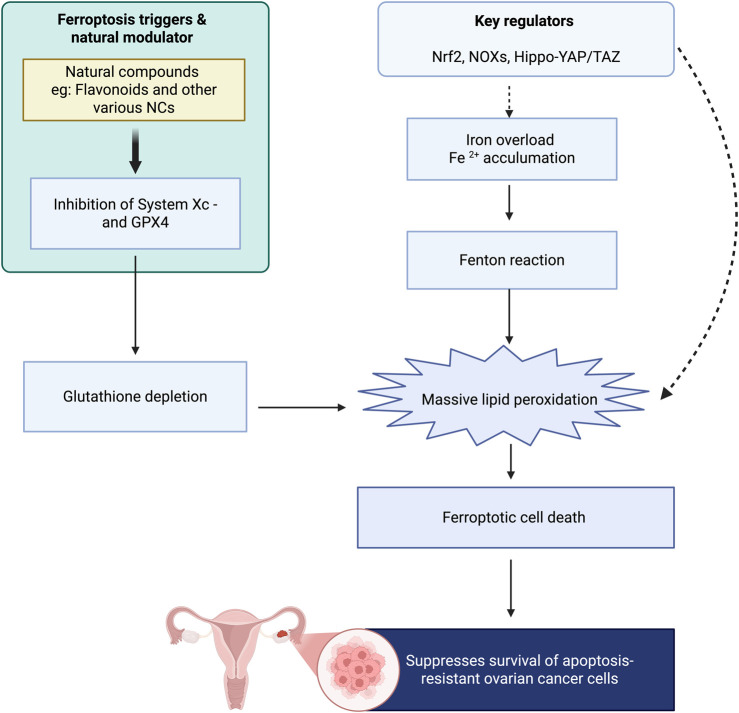
Key knowledge gaps and promising future directions for targeting ferroptosis in OC.

Ferroptosis is an iron-catalyzed, regulated form of cell death characterized by lipid peroxidation and excessive production of ROS and has emerged as a promising therapeutic target for OC in recent times (Zhao H. et al., 2022). Unlike apoptosis or autophagy, ferroptosis is morphologically separable with contracted mitochondria, compact membranes, reduced cristae, but a preserved plasma membrane (Tang et al., 2021). In OC cells, ferroptosis is regulated primarily by lipid peroxidation and iron metabolism: increased iron uptake via transferrin receptor 1 (TFR1), labile iron pool accumulation of ferrous iron (Fe^2+^), and aberrant regulation of ferritin and ferroportin create a pro-oxidant condition that promotes lipid ROS generation by Fenton chemistry (Liu D. et al., 2024). Polyunsaturated fatty acid-phospholipid substrates of lipid peroxidation, when uncontrolled, cause ferroptotic cell death. Key regulators include glutathione peroxidase 4 (GPX4), which decreases lipid peroxides, and the cystine/glutamate antiporter System Xc^−^, which sustains glutathione synthesis to supply GPX4; inhibition of either pathway triggers ferroptosis. Additional modulators include nuclear factor erythroid 2-related factor 2 (Nrf2), which enhances antioxidant defenses and guards against ferroptosis, and NADPH oxidases (NOXs), which enhance ROS accumulation (Chen et al., 2024). Mitochondria are also involved through ROS production and structural alterations that increase ferroptotic reactions, and susceptibility is also regulated by the Hippo–YAP/TAZ pathway, with TAZ-induced upregulation of ANGPTL4 activating NOX2 and increasing lipid peroxidation (Yang and Chi, 2019). One exciting new frontier in the action of natural products is that they have the ability to cause ferroptosis by causing iron overload and lipid peroxidation, or through the modulation of glutathione metabolism and inhibition of GPX4, thus opening a promising direction to target chemoresistant OC cells with an apoptotic-resistant phenotype (Na et al., 2024; Al Amin et al., 2025; Zhao et al., 2025) (Figure 2). Although still in the discovery phase, this mechanism represents an encouraging addition to the anticancer armory of NCs. Meanwhile, huge areas of ignorance remain: little direct evidence exists for NC ferroptosis induction in OC, most studies rely on preclinical models with no clinical translation, and interactions between ferroptosis and other mechanisms of death such as apoptosis, autophagy, and immunogenic cell death are unclear. Furthermore, how much the damage-associated molecular patterns produced by ferroptosis (HMGB1) impact the tumor immune microenvironment remains to be discovered, and how OC cells gain resistance against ferroptosis possibly via Nrf2 activation, lipid remodeling, and altered iron management are just beginning to be unveiled. These gaps place ferroptosis into the limelight as a promising but underemphasized vulnerability in OC biology that requires additional mechanistic investigation and therapeutic targeting, particularly in combination with chemotherapy, radiotherapy, immunotherapy, or natural products that have the potential to serve as ferroptosis modifiers.

## 4 Selected example of NCs as chemo preventive drugs against OC

### 4.1 Voacamine

Voacamine (VOA), an indole alkaloid from the Apocynaceae family (*Voacanga* and *Tabernaemontana* genera), exhibits notable chemosensitizing potential in drug-resistant OC (Wu et al., 2021; Condello et al., 2020; Pellegrini et al., 2022). Although minimally cytotoxic to normal cells, VOA enhances the efficacy of chemotherapeutic agents such as paclitaxel (PTX) and doxorubicin (DOX), particularly in multidrug-resistant OC cell lines (A2780 DX). Its primary mechanism involves inhibition of P-glycoprotein (P-gp), an efflux transporter responsible for reduced intracellular drug accumulation in resistant cells (Zou et al., 2024). By blocking P-gp function, VOA restores intracellular PTX and DOX levels, leading to enhanced apoptosis and G2/M cell cycle arrest (Zou et al., 2024; Chai et al., 2010). Additionally, VOA disrupts NF-κB nuclear translocation, suppressing downstream anti-apoptotic signaling and further promoting chemosensitivity (Karthika et al., 2022; Nath et al., 2025). Importantly, VOA demonstrates low toxicity to normal cells at effective concentrations, suggesting a favorable therapeutic window for combination therapy. Preliminary evidence also indicates its potential antiangiogenic effects via inhibition of HIF-1α and VEGF, though this remains to be fully validated in OC models (Kim et al., 2012; Huang et al., 2019).

### 4.2 Resveratrol

Resveratrol is a natural polyphenol found in abundance in grapes, red wine, berries, and peanuts, which has attracted considerable attention due to its wide-ranging anticancer activity against OC (Xu et al., 2021). It is of low toxicity, is available through diet, and exhibits multi-targeted molecular action, thus representing a promising candidate for integrative cancer therapy. Resveratrol acts through various antitumor means in OC by promoting apoptosis, inhibiting proliferation, affecting metabolic pathways, and reducing inflammation that make cancer cells sensitive to chemotherapy (Chitcholtan et al., 2024). It increases intracellular ROS, thus causing oxidative stress, which triggers mitochondrial apoptosis by releasing cytochrome c and activating caspases. Resveratrol, in the cell lines A2780 and SKOV3 of OC, suppressed viability and enhanced apoptosis by downregulating the Notch1/PTEN/Akt axis, an important pathway involved in cancer stemness and survival (Kim et al., 2019).

Moreover, resveratrol impairs glucose metabolism by inhibiting Akt/mTOR phosphorylation, reducing glucose uptake, lactate production, and glycolytic flux, thus disrupting the “Warburg-like” metabolic phenotype essential for tumor growth (Brockmueller et al., 2021). It also induces autophagy that cooperates with apoptosis for the eradication of resistant tumor cell populations. It modulates the tumor microenvironment by inhibiting NF-κB–mediated inflammation and preventing angiogenesis due to downregulation of HIF-1α and VEGF, therefore limiting tumor invasion and metastasis (Xu et al., 2021). Importantly, it increases chemosensitivity, in particular to cisplatin, by inhibiting epithelial–mesenchymal transition and lowering the apoptotic threshold in resistant OC models (Baribeau et al., 2014). Together, the regulation of apoptosis, metabolic pathways, autophagy, inflammation, and angiogenesis makes resveratrol a promising agent for chemosensitization in ovarian cancer.

### 4.3 Quercetin

Quercetin is one of the most abundant dietary flavonoids found in apples, onions, berries, and leafy greens. It has gained significant interest due to its broad-spectrum anticancer potential, including against OC (Aghababaei and Hadidi, 2023). Low toxicity, accessibility, and multi-targeted mechanisms make this agent a potential adjunctive therapeutic agent. Preclinical studies reveal that quercetin exerts its anti-OC effects by inhibiting the growth of OC cells, inducing apoptosis, suppressing metastasis, modulating oxidative stress, and reversing chemotherapy resistance (Vafadar et al., 2020). Quercetin induces cell cycle arrest at the G0/G1 phase, which halts DNA synthesis and mitosis in OC cell lines such as SKOV-3, A2780, and OVCAR-3. It also activates mitochondrial-mediated apoptosis through downregulation of anti-apoptotic proteins (Bcl-2, survivin) and upregulation of pro-apoptotic markers (Bax, Bid, cytochrome c, and caspases 3, 8, and 9) (Ren et al., 2015; Catanzaro et al., 2015). Quercetin exerts dual redox effects: it enhances antioxidant enzymes such as superoxide dismutase (SOD1) to maintain a proper redox balance, and under stress conditions, it acts as a pro-oxidant by enhancing the apoptosis of cancer cells (Alharbi et al., 2025; Biswas et al., 2022). It inhibits metastasis by downregulating matrix metalloproteinases (MMP-1, MMP-2, and MMP-9) and suppressing epithelial-mesenchymal transition (EMT) by inhibiting transcription factors Snail and Twist, ultimately reducing invasion and recurrence (Dhanaraj et al., 2021).

One of the significant therapeutic benefits of quercetin is its chemoresistance-overcoming properties. It downregulates P-glycoprotein (P-gp/ABCB1), which enhances intracellular accumulation and increases the efficacy of chemotherapeutic agents such as cisplatin, paclitaxel, and doxorubicin (Maciejczyk and Surowiak, 2013). When administered in combination with paclitaxel, quercetin synergistically promotes apoptosis and inhibits survival pathways, including PI3K/Akt, STAT3, and NF-κB, leading to enhanced cytotoxicity with a potentially reduced drug dosage and systemic toxicity (Silva-Pinto et al., 2025). Collectively, the multi-modal actions of quercetin on apoptosis, redox balance, metastasis, and drug resistance underpin its potential as a complementary approach in ovarian cancer therapy.

### 4.4 Epigallocatechin-3-gallate

Epigallocatechin-3-gallate (EGCG), the most bioactive catechin in green tea, is a potent natural anticancer agent with multiple molecular targets against OC (Talib et al., 2024). Due to good safety and low toxicity, it has been investigated for single-agent and combination therapies. Robust *in vitro* and *in vivo* evidence points to its potential for inhibiting tumor growth, modulating several signaling pathways, inducing apoptosis, and overcoming drug resistance in OC cells (Kciuk et al., 2023). Treatment of EGCG significantly repressed the proliferation of OC cell lines such as SKOV3 and OVCAR-3 in a dose-dependent manner (Jin et al., 2015; Zhang et al., 2025). Intraperitoneal EGCG, 50 mg/kg, in xenograft mice reduced tumor volume by >70%, superior to paclitaxel’s effect, without toxicity or weight loss (Rodriguez Torres et al., 2023). The molecular mechanism underlying this involves G1-phase arrest and apoptosis mediated through upregulation of p21 and Bax and downregulation of cyclin D1, CDK4, and Bcl-xL (Kim et al., 2004). PTEN/AKT/mTOR pathway targeting by EGCG activates PTEN and inhibits AKT/mTOR phosphorylation to suppress oncogenic signaling (Qin et al., 2020). EGCG also exhibited modifications in the endothelin-1/ET(A) axis, reducing COX-1, COX-2, and PGE2 expression, thereby limiting the inflammatory angiogenic cascade (Almatroodi et al., 2020). More importantly, EGCG sensitizes chemoresistant OC cells to cisplatin and paclitaxel by inhibiting drug efflux and reversing EMT-related and modulating apoptosis-related genes (Almatroodi et al., 2020; Włodarczyk et al., 2024). Antioxidant and anti-inflammatory effects of EGCG also augment the chemosensitivity of these cells further (Capasso et al., 2025). Although EGCG exhibits excellent safety even at very high dosages, its clinical utility is limited due to poor bioavailability and rapid metabolism. Strategies to improve its pharmacokinetics and thus its therapeutic effect include nanoparticle or liposomal encapsulation and EGCG analogs (Alam et al., 2024; Wong et al., 2025).

### 4.5 β-Escin

β-escin is a saponin derived from horse chestnut (*Aesculus hippocastanum*), known for its surface-active properties (Geisler et al., 2020). This compound has been utilized in medical and cosmetic applications due to its anti-inflammatory, anti-edematous, and vasoprotective characteristics (Gallelli, 2019). In medical settings, β-escin has been studied for its therapeutic potential in treating chronic venous insufficiency, varicose veins, and haemorrhoids (Fa et al., 2023), as well as for its emerging roles in cancer therapy, particularly due to its anti-proliferative and pro-apoptotic effects (Kenny et al., 2021). β-escin shows therapeutic promise in OC by inducing apoptosis in A2780 cells through increased oxidative stress, mitochondrial membrane potential disruption, and G2/M cell-cycle arrest (Cheong et al., 2018). It also suppresses the p38 MAPK/ERK signalling pathway, reduces metastasis, and limits cell adhesion to the TME, possibly by impacting HIF1α pathways (Zhao W. et al., 2022). Additionally, β-escin inhibits Na+,K + -ATPase, which may disrupt cancer cell adhesion and trigger apoptosis, resembling the action of cardiac glycosides (Fa et al., 2023). These effects are more pronounced in human cells than in mouse cells, suggesting species-specific differences. Further studies are needed to explore β-escin’s broader mechanisms and its potential as a cancer therapy.

### 4.6 Curcumin

Turmeric or *Curcuma longa* is a polyphenolic compound with strong anti-inflammatory, antioxidant, and anticancer activities (Sharifi-Rad et al., 2020). In OC, curcumin acts upon modulation of proliferation, apoptosis, cell cycle, remodeling of the tumor microenvironment, and chemosensitization in both *in vitro* and *in vivo* models (Zahra et al., 2022). Although the low bioavailability of curcumin has precluded its clinical use, recent advances in nanoformulations are aimed at enhancing its therapeutic potential. Curcumin suppressed viability and colony formation in a doseand time-dependent manner in SKOV3, A2780, and OVCAR3 cells through the upregulation of active caspase-3 and Bax, and downregulation of PCNA (Sun and Fang, 2021). More significantly, curcumin has been shown to significantly reduce tumor size in xenograft models of OC (Akter et al., 2025). Mechanistically, epigenetic modifications mediated by non-coding RNAs, including upregulation of circ-PLEKHM3 that sponges oncogenic miR-320a, leading to increased expression of SMG1, which commits the cells to apoptosis, have been implicated (Sun and Fang, 2021). Its antiproliferative effects are partly due to induction of G2/M arrest caused by downregulation of cyclin B1 and CDC25A, which suppresses mitosis (Liu et al., 2023). Treatment of OC cells with curcumin results in increased levels of ROS, leading to mitochondrial dysfunction, depolarization of the mitochondrial membrane, and apoptosis, which are reversed by antioxidants, indicating oxidative stress as the key cytotoxic mechanism (Hu et al., 2023). Furthermore, curcumin modulates the tumor immune microenvironment by promoting M1 and suppressing M2 macrophage phenotypes, reducing EMT, invasion, and migration (Parker et al., 2025). Notably, curcumin promotes the chemosensitivity of OC cells by suppressing NF-κB signaling and upregulating SNIP1, thus sensitizing resistant OC cells to cisplatin and doxorubicin (Cacciola et al., 2023). Additionally, curcumin inhibits β-catenin/TCF and AKT/mTOR pathways, reducing stemness, EMT, survival, and metabolism (Akter et al., 2025).

### 4.7 Berberine

Berberine, an isoquinoline alkaloid from *Berberis vulgaris*, *Coptis chinensis*, and *Hydrastis canadensis*, has been shown to present broad-spectrum anticancer properties (Rauf et al., 2021). In the context of OC, this natural product inhibits cell growth, triggers apoptosis, increases chemosensitivity, affects epigenetic regulation, and interferes with DNA repair pathways. Berberine reduces proliferation in SKOV3, A2780, and OVCAR3 cells in a dose- and time-dependent mode, triggers apoptosis via both intrinsic and extrinsic pathways through downregulation of BCL-2 and survivin and upregulation of BAX, leading to caspase-3 activation and PARP cleavage (Almatroodi et al., 2022). These events further induce necroptosis, enhancing tumor cell death. Combination of berberine with cisplatin or paclitaxel synergistically induces G0/G1 arrest, caspase activation, and apoptosis, leading to stronger inhibition of tumors, which might facilitate reduced doses of chemotherapies and reduced toxicity (Liu L. et al., 2019).

Epigenetically, it reactivates, through promoter demethylation, the DNA mismatch repair gene hMLH1, which consequently restores genomic stability (Jin et al., 2015). It suppresses migration and invasion by upregulating miR-145, which targets the downregulation of MMP16, and by weakening lipid metabolism associated with metastasis (Zhang et al., 2025). Berberine disrupts glycolysis and lactate production, opposing the Warburg effect, and inhibits autophagy through the LINC01123/P65/MAPK10 axis, reducing the survival and dissemination of cancer cells (Yan et al., 2024). Besides, it triggers oxidative DNA damage and abrogates homologous recombination repair through the downregulation of RAD51, thus sensitizing cells to the PARP inhibitor niraparib, which particularly exerts a high efficacy in BRCA-wildtype OC (Hou et al., 2017). Furthermore, berberine overcomes chemoresistance by inhibiting oncogenic miRNAs such as miR-93 and restoring drug sensitivity and reducing tumor growth (Farooqi et al., 2019). Collectively, its multitargeted action and synergistic potential make berberine an attractive adjuvant in OC therapy.

### 4.8 Sulforaphane

Sulforaphane, an isothiocyanate from cruciferous vegetables like broccoli and kale, shows promise in fighting various cancers, including cervical, breast, bladder, renal, lung, colon, and prostate cancers (Kaiser et al., 2021). It enhances the effectiveness of chemo-therapeutic agents like cisplatin by reversing resistance, as seen in OC cell lines like A2780/CP70 and IGROV1-R10 (Ullah, 2015). Sulforaphane induces DNA damage, increases intracellular cisplatin, and boosts miR-30a-3p expression, which is typically lower in cisplatin-resistant cells (Gong et al., 2020). Sulforaphane also inhibits OC cell growth through redox mechanisms, activating anticancer responses like p53 and ARE while suppressing tumor-promoting pathways such as AP-1 and HIF-1. By reducing HIF-1α protein levels, sulforaphane impacts CA IX, lowering tumor cell migration and improving chemo-therapy sensitivity (Otoo and Allen, 2023). Despite its benefits, thiol-reducing agents can negate sulforaphane’s anti-cancer effects, suggesting they should not be combined with sulforaphane-based treatments (Kim et al., 2017). Overall, sulforaphane has significant potential in overcoming chemoresistance in OC.

### 4.9 Ellagic acid

Ellagic acid (EA) is a naturally occurring polyphenol present in berries, pomegranates, and nuts that exerts broad anticancer activities in the form of anti-proliferative, pro-apoptotic, antiinflammatory, and anti-metastatic effects (Čižmáriková et al., 2023). In OC, EA is a multi-target agent with efficacy as a monotherapy and/or chemosensitizer, in particular in chemoresistant and CSC populations (Chung et al., 2013). EA suppresses proliferation of OC cell lines ES-2, PA-1, A2780 and OVCAR3 by inducing G1-phase arrest via increased expression of p53/p21 along with downregulating cyclins D1 and E (Chung et al., 2013). The promotion of apoptosis is evidenced by the increased Bax/Bcl-2 ratio, caspase-3 activation, and DNA fragmentation. EA reactivates anoikis, hence impending anchorage-independent growth and metastasis (Zhan et al., 2014).

Further, it suppresses pyroptosis and inflammation through downregulation of gasdermin D/E and reduces IL-1β/IL-6, thereby modulating the tumor-promoting microenvironment (Sun et al., 2025). It acts via targeting CSCs by impairing self-renewal, increasing ROS, disrupting mitochondrial function, and inducing apoptosis (Mandal et al., 2024). EA enhances cisplatin sensitivity by inhibiting DNA repair and promoting apoptosis and hence appears to exhibit strong chemosensitizing potential (Mandal et al., 2024). Moreover, EA inhibits invasion and metastasis through suppression of MMP2 and MMP9 expressions, reducing extracellular matrix degradation and metastatic spread (Bindhu et al., 2025). Overall, these multi-mechanistic effects make EA a promising adjuvant candidate in OC therapy.

### 4.10 Withaferin

Withaferin A (WFA) is a bioactive steroidal lactone isolated from *Withania somnifera* (ashwagandha) that has exhibited potent anticancer activity in preclinical OC models (Kumar et al., 2023). Unlike conventional agents targeting bulk tumor cells, WFA eliminates CSCs, which cause chemoresistance, relapse, and metastasis. WFA, as a single agent or in combination with cisplatin, reduced the tumor burden by 70%–80% and completely prevented distant metastasis in the orthotopic OC mouse model (Kakar et al., 2014). While treatment with cisplatin alone enriched CSCs, WFA suppressed them by downregulating the stemness markers of CD44, CD24, CD34, CD117, and Oct4 and inhibiting Notch signaling factors of Notch1, Hes1, and Hey1, thereby preventing relapse and resistance (Sultana et al., 2021).

Beyond CSC targeting, WFA induces G2/M cell cycle arrest by upregulating p21 and downregulating cyclin B1 and Aurora kinases, leading to mitotic catastrophe and growth inhibition (Lee and Choi, 2016). It also triggers caspase-3, suppresses Bcl-2, and enhances mitochondrial membrane permeabilization, thereby inducing apoptosis. WFA acts anti-metastatically through downregulation of MMP-2, MMP-9, vimentin, and N-cadherin and by suppressing VEGF-mediated angiogenesis (Xing et al., 2023). These effects are partly mediated through the inhibition of PI3K/Akt and TGF-β/Smad2 pathways, known key drivers of OC progression and immune evasion.

Besides that, WFA also exerts non-apoptotic death forms, including autophagy and ferroptosis to kill apoptosis-resistant cells 182. It further exerts immune-modulatory effects by reducing TNF-α, IL-6, and NF-κB activity, thus favoring an anti-tumor immune milieu, which further increases the responsiveness of chemotherapy and immunotherapy (Mohan et al., 2004). Collectively, WFA’s multi-targeting action against CSCs, proliferation, metastasis, and inflammation justifies its potential as a novel therapeutic candidate in OC.

### 4.11 Celastrol

Celastrol, a quinone methide triterpene from *Tripterygium wilfordii*, has been reported as a potent natural anticancer agent with broad efficacy against multiple malignancies, including OC (Shi et al., 2020). Both *in vitro* and *in vivo* studies in the OC model indicate that celastrol shows very good selectivity to cancer cells with low toxicity against normal cells, manifesting anti-proliferative, pro-apoptotic, anti-inflammatory, and anti-metastatic effects (Shi et al., 2020; Wang et al., 2023). It has been found to induce apoptosis by upregulating cleaved PARP and activating p38 MAPK and JNK while inhibiting ERK and PI3K/Akt pathways (Ma Y. T. et al., 2025). Besides, the G2/M cell cycle arrest resulting from modulation of p27, Cyclin B1, and Cyclin E impairs growth and enhances cell death. Bioinformatic systems biology analyses identified MYC, CDC37, and FN1 as key targets, implicating celastrol in DNA repair inhibition and disruption of replication processes. It also suppresses NF-κB and JAK2/STAT3 signaling, reducing IL-6 and other pro-inflammatory cytokines (Zhu et al., 2024). Anti-metastatic effects occur through the inhibition of MMPs and EMT via NF-κB and MAPK suppression. *In vivo*, celastrol significantly reduces tumor burden with minor toxicity (Wang et al., 2022). Advances in formulation are improving the bioavailability and therapeutic index of celastrol, including celastrol-loaded nanoparticles, liposomes, and targeted analogs.

### 4.12 Others

Fucosterol, a phytosterol from algae and seaweed, exhibits anticancer potential in a wide range of cancers, including OC (Meinita et al., 2021). It triggers ER stress and mitochondrial dysfunction, culminating in cell cycle arrest in OC cells and inhibits tumor growth by activating ER stress sensors, while concurrently inhibiting the PI3K/AKT/mTOR pathway (Bae et al., 2020). It also suppresses angiogenesis-related genes to reduce vascularization and tumor progression (Bae et al., 2020; Singla et al., 2022). Zerumbone, isolated from *Zingiber zerumbet*, induces apoptosis and G2/M arrest in OC and cervical cancer cells, downregulates IL-6 secretion, and increases chemosensitivity (Abdelwahab et al., 2012). St. John’s wort *Hypericum perforatum* via its bioactive hypericin can alter the cytotoxicity of anticancer drugs by modifying ABC transporters—lowering the efficacy of cisplatin and mitoxantrone while increasing MTX-induced death in BCRP-overexpressing cells, highlighting its complex drug interactions (Jendželovská et al., 2014). Kombucha-fermented extract (KFE) encapsulated in PLGA nanoparticles selectively targets A2780 OC cells and encourages apoptosis and inhibits angiogenesis by VEGF suppression (Ghandehari et al., 2023). Tanshinone I (Tan-I) from *Salvia miltiorrhiza* enhances the efficacy of paclitaxel, promoting apoptosis, DNA damage, and senescence *in vitro* and *in vivo* (Zhou et al., 2020). Pomegranate peel extract (PPE) impairs the metabolic activity of OC cells, increases oxidative stress, and decreases growth factor expression in OVCAR-3 cells and has shown antioxidant activity in normal granulosa cells (Kolesarova et al., 2023). Similarly, Tagetes erecta petal extract has been found to have high antioxidant and pro-apoptotic efficiency against OC cells (Rivas-García et al., 2023).

Despite robust preclinical evidence supporting natural compounds in OC, clinical translation remains limited. Many phytochemicals (quercetin, curcumin, resveratrol, berberine) exhibit multi-target antitumor effects—apoptosis induction, autophagy modulation, proliferation inhibition, and chemoresistance reversal—but human trials are scarce (Lu et al., 2025b). Quercetin enhances cisplatin and radiation sensitivity through p53 activation (Vafadar et al., 2020), while ongoing trials, such as NCT05306002, are assessing curcumin’s adjuvant potential in OC. Clinical advancement is hindered by low bioavailability, poor solubility, and metabolic instability (Vrânceanu et al., 2022). Moreover, natural products are often categorized as supplements rather than therapeutic agents, slowing their inclusion in oncology pipelines. However, integrative oncology is increasingly investigating NCs as synergistic partners with standard therapies to improve efficacy and reduce toxicity (Berretta et al., 2025). Emerging studies on semi-synthetic derivatives and multi-component herbal formulations show promise but often lack standardization and rigorous design (Islam et al., 2023).

Overall, NCs such as alkaloids, flavonoids, polyphenols, terpenoids, and isothiocyanates have shown potent chemopreventive and chemosensitizing effects in OC through a variety of mechanisms, affecting important pathways like PI3K/Akt/mTOR, NF-κB, Wnt/β-catenin, and MAPK (Table 1). Although preclinical data are promising (Table 2), future success relies on enhancement in the areas of bioavailability, formulation, and clinical validation by well-designed trials.

**TABLE 1:** The natural compounds and their mechanisms of action against OC.

Compound	Source	Primary mechanisms of action	Key effects on OC	Combination benefits
Voacamine (VOA)	*Voacanga*, *Tabernaemontana* spp.	P-glycoprotein inhibition; NF-κB translocation disruption; HIF-1α/VEGF inhibition	Enhances drug accumulation; G2/M arrest; apoptosis via PARP1 cleavage	Synergistic with paclitaxel, doxorubicin in MDR cell lines
Resveratrol	Grapes, berries, peanuts	ROS generation; Notch1/PTEN/Akt inhibition; NF-κB and EMT suppression	Apoptosis; autophagy; anti-angiogenesis; metabolic disruption	Enhances cisplatin sensitivity; overcomes resistance
Quercetin	Apples, onions, leafy greens	P-gp downregulation; MMP/EMT suppression; antioxidant enzyme induction	G0/G1 arrest; mitochondrial apoptosis; inhibits invasion and migration	Synergizes with cisplatin, paclitaxel, doxorubicin
EGCG	Green tea	PTEN/AKT/mTOR modulation; p21↑, cyclin D1↓; ET-1 receptor inhibition	Tumor reduction; G1 arrest; Bax↑, Bcl-xL↓; enhanced apoptosis	Sensitizes to cisplatin and paclitaxel
β-Escin	*Aesculus hippocastanum* (chestnut)	Na^+^/K^+^-ATPase inhibition; p38 MAPK/ERK suppression; HIF-1α pathway modulation	Oxidative stress-mediated apoptosis; G2/M arrest; reduced metastasis	Potential synergy with cardiac glycosides
Curcumin	*Curcuma longa* (turmeric)	Epigenetic modulation; ROS generation; NF-κB/SNIP1 regulation; macrophage polarization	G2/M arrest; mitochondrial dysfunction; tumor microenvironment and EMT modulation	Enhances cisplatin, doxorubicin effects; reverses drug resistance
Berberine	*Berberis*, *Coptis*, *Hydrastis* spp.	hMLH1 demethylation; RAD51↓; metabolic reprogramming; miRNA modulation	Apoptosis; necroptosis; G0/G1 arrest; synthetic lethality with PARPi	Synergistic with cisplatin, paclitaxel; enhances PARPi (e.g., niraparib) response
Sulforaphane	Broccoli, cruciferous vegetables	DNA damage induction; miR-30a-3p↑; HIF-1α↓; redox activation	Reverses cisplatin resistance; increases drug uptake; p53 and ARE activation	Enhances cisplatin in resistant lines (A2780/CP70, IGROV1-R10)
Ellagic acid	Berries, pomegranates, nuts	p53/Cip1↑; Bax/Bcl-2 modulation; anoikis reactivation; MMP2/9 suppression	G1 arrest; mitochondrial apoptosis; anti-metastatic; CSC targeting	Synergistic with cisplatin; enhances DNA damage
Withaferin A	*Withania somnifera* (ashwagandha)	CSC inhibition; Notch/PI3K/Akt/TGF-β/Smad2 suppression; multiple cell death pathways	Tumor burden reduction (70%–80%); metastasis elimination; G2/M arrest; mitotic catastrophe	Reverses cisplatin-induced CSC enrichment; effective in resistant models
Celastrol	*Tripterygium wilfordii*	MYC/CDC37/FN1 binding; NF-κB and JAK2/STAT3 inhibition; DNA repair impairment; MAPK regulation	G2/M arrest; p38 MAPK/JNK activation; MMP downregulation; anti-metastasis	Multi-target synergy; candidate for nanoformulation
Fucosterol	Seaweed, algae	ER stress induction; PI3K/AKT/mTOR inhibition; angiogenesis gene downregulation	Cell cycle arrest; mitochondrial dysfunction; angiogenesis inhibition	Shown effective in zebrafish xenografts
Zerumbone	*Zingiber zerumbet* (ginger)	G2/M arrest; IL-6↓; apoptosis induction	Growth suppression; chemo-sensitization	IL-6 suppression improves chemo-response
Hypericin	*Hypericum perforatum* (st. John’s wort)	ABC transporter modulation; drug-resistance interaction	Variable effects; may interfere with cisplatin or enhance other drugs	Use caution—may reduce cisplatin efficacy; context-dependent
Tanshinone I	*Salvia miltiorrhiza*	DNA damage potentiation; senescence promotion; apoptosis enhancement	Reduced proliferation; treatment efficacy ↑	Synergistic with paclitaxel in A2780 and ID-8 models

**TABLE 2:** Evidence strength of NCs in OC.

Compound	*In vitro* evidence	*In vivo* models	Early clinical studies	Overall evidence strength
Curcumin	✓✓✓	✓✓✓	—	Strong
Resveratrol	✓✓✓	✓✓	—	Strong/Moderate
Quercetin	✓✓✓	✓✓	—	Moderate
Berberine	✓✓✓	✓	—	Moderate
Sulforaphane	✓✓	✓	—	Moderate
EGCG	✓✓✓	✓✓	—	Moderate
Withaferin A	✓✓✓	✓✓	—	Moderate
Celastrol	✓✓✓	✓	—	Moderate
β-Escin	✓	—	—	Limited
Ellagic acid	✓	—	—	Limited
Others (fucosterol, zerumbone, pomegranate extract)	✓	Occasional	—	Preliminary

## 5 Biomedical technologies and delivery systems for NCs in OC

The clinic translation of the NCs in OCis hindered by various challenges, but low bioavailability and adverse pharmacokinetics remain the biggest limiting factors. Despite their robust multitarget anticancer activities, curcumin, quercetin, resveratrol, and EGCG are characterized by poor aqueous solubility, poor intestinal absorption, extensive metabolism, and systemic clearance (Chimento et al., 2023). These limitations exclude traceability to therapeutic levels in tumor tissue and undermine consistency between preclinical efficacy and clinical response. While irregularity in natural compounds’ composition, regulatory difficulties, and reproducibility are equally troublesome, they are considered secondary to pharmacokinetic limitations, which present the principal bottleneck for successful clinical translation (Asiri et al., 2025).

Advances in biomedical technologies have substantially improved the therapeutic efficacy of NCs against OC by overcoming such major weaknesses, primarily poor bioavailability, short lifespan, decreased tumor targeting, and drug resistance (Zeinali et al., 2025). Among such technologies, NDDS on a nanoscale has been extensively explored because they have the ability to encapsulate NCs for their improved solubility, stability, and targeted release (Liu Y. et al., 2024). NDDS such as nanoparticles, liposomes, micelles, dendrimers, nanocapsules, and NLCs possess the benefits of controlled release, reduced systemic toxicity, and enhanced accumulation at cancer sites by the passive (EPR effect) or active targeting mechanisms (Pantshwa et al., 2020).

Polymeric nanoparticles are a novel approach to improving NC delivery in OC with potential to counteract pharmacokinetic and pharmacodynamic constraints such as poor solubility, low stability, and lack of sufficient tumor specificity (Elmowafy et al., 2023). By encapsulating phytochemicals in biocompatible polymeric matrices, nanoparticles favor slow release, enhanced bioavailability, and enhanced therapeutic targeting, all of which are critical factors in the treatment of as complex and drug-resistant an illness as OC (Gralewska et al., 2024). Encapsulating curcumin and other metabolites in hydrophilic polymers such as PHEMA or PLGA enhances anticancer activity (Pandey et al., 2025), suppressing tumor growth and modulating apoptotic and angiogenic markers (Bax, Bcl-2, survivin, VEGF, COX-2) and also signaling pathways such as NF-κB, JAK/STAT3, and PI3K/Akt (Zahra et al., 2022; Yan et al., 2025). Importantly, polymeric nanoparticles also bypass drug efflux pumps, resensitizing drug-resistant OC cells (A2780CP), thereby overcoming the biggest barrier of multidrug resistance (Levit and Tang, 2021). Active targeting approaches with folic acid, RGD peptides, or monoclonal antibodies also increase tumor specificity (Muhamad et al., 2018). The latest advances even allow co-delivery of nucleic acids (siRNA) along with phytochemicals, diversifying therapeutic modulation of oncogenic pathways (Muhamad et al., 2018; You and Zhang, 2025).

Liposomes are amongst the most clinically validated nanocarriers for NC delivery in OC (Nsairat et al., 2022). These lipid vesicles encapsulate hydrophilic and lipophilic drugs, improving solubility, stability, and bioavailability (Kumar G. et al., 2024). Their intrinsic accumulation in tumors via the EPR effect and design suitability for ligand-mediated active targeting make them highly versatile (Abbasi et al., 2023). PEGylation delays circulation time and ligand-functionalized liposomes enhance tumor specificity (Taher et al., 2023). Liposomal chrysin, for instance, was more anti-proliferative and pro-apoptotic in SKOV-3 cells than free chrysin (Tarahomi et al., 2023). Similarly, curcumin, resveratrol, and quercetin in liposomal formulations displayed superior pharmacokinetics, reduced tumor load, and enhanced therapeutic efficacy in preclinical models (Trofin et al., 2025).

Dendrimers possess strict structure control, high loading capacity, and versatile surface modification (Chis et al., 2020). PAMAM dendrimers are highly effective, enabling NCs such as curcumin and quercetin encapsulation, thereby improving solubility, stability, and cellular incorporation (Alamos-Musre et al., 2025). Their ability to incorporate tumor-targeting ligands (folic acid, RGD peptides, AS1411 aptamer) enhances selective delivery (Pérez-Ferreiro et al., 2023). Stimulus-responsive dendrimers releasing drugs in acidic tumor microenvironments or under enzymatic activation further increase therapeutic precision. Significantly, hybrid lipid–dendrimer nanoparticles showed >30-fold improved activity over free paclitaxel, suggesting the promise of hybrid systems (Liu et al., 2015).

Micelles are colloidal self-associated carriers with hydrophobic cores ideal for delivering poorly soluble NCs. They enhance stability, prolong circulation time, and protect against degradation (Hanafy et al., 2018). RGD-functionalized tumor-targeted micelles exhibited improved integrin-positive OC cell uptake, arresting growth and angiogenesis in the tumor (Javid et al., 2024). Micellar formulations of fisetin, ellagic acid, and shikonin exhibited greatly improved induction of apoptosis and anti-metastatic activity against OC models (Xiao et al., 2018).

Nanocapsules containing a drug-loaded core and a polymeric coat protect NCs against premature degradation while enabling controlled release and targeting to tumors (Deng et al., 2020). Folic acid ligand functionalization enhances specificity, while encapsulation enhances drug stability and tumor accumulation. As an example, cisplatin-loaded PEG nanocapsules showed ∼90% inhibition of xenograft tumors, which is better than free cisplatin (Jurczyk et al., 2021). Nanocapsules also overcome multidrug resistance by efflux pump bypass, as shown in the case of paclitaxel–lapatinib co-loaded nanocapsules that synergistically inhibited resistant OC cells (Saripilli and Sharma, 2025).

Nanostructured lipid carriers (NLCs) consist of solid–liquid lipid mixtures and have high drug-loading capacity, stability, and prolonged release (Khan et al., 2023). NLCs are particularly advantageous for hydrophobic NCs such as silibinin and maximize encapsulation, stability, and cell uptake (Viegas et al., 2023). NLCs also reverse chemoresistance, as seen in cisplatin-resistant A2780CP cells where cisplatin–silibinin co-loaded NLCs potently inhibited growth (Jafari et al., 2022). Oral NLCs are in development to improve patient compliance and avoid hepatic first-pass metabolism (Elmowafy and Al-Sanea, 2021). Mechanistically, NLCs promote intracellular delivery of NCs, induce apoptosis, and inhibit PI3K/Akt, MAPK, and VEGF pathways, while evading efflux transporter action (Waheed et al., 2024). A summary of nanodelivery platforms used to enhance the anticancer efficacy of NCs in OC therapy provided in Table 3.

**TABLE 3:** Nanodelivery systems for enhancing NCs efficacy in OC.

Delivery system	Structure/Composition	Key advantages	Natural compounds delivered	Mechanisms of enhancement	Clinical benefits	Targeting strategies
Polymeric nanoparticles	PHEMA, PLGA matrices; biocompatible polymers; controlled release systems	Improved solubility and stability; controlled release; cellular internalization	Curcumin, various phytochemicals; co-delivery with siRNA	Bypass efflux pumps; ↑ intracellular accumulation; modulate NF-κB, JAK/STAT3, PI3K/Akt; ↑ Bax, ↓ Bcl-2	Overcome MDR (e.g., A2780CP); potent apoptosis; gene silencing	Folic acid, RGD peptides, monoclonal antibodies
Liposomes	Spherical bilayer vesicles; phospholipid membranes; load hydrophilic/hydrophobic compounds	EPR accumulation; protection from degradation; clinical validation	Chrysin, curcumin, quercetin, resveratrol	↑ cellular uptake; ↑ Bax, caspase-3; ↓ Bcl-2; signaling pathway suppression	Tumor burden reduction; improved pharmacokinetics; minimal systemic toxicity	PEGylation; folate receptor-α, LHRH receptor, and CD44 targeting
Dendrimers	PAMAM, PPI, PLL; central core with branched arms; terminal functional groups; monodisperse macromolecules	High loading capacity; modifiable structure; responsive release	Quercetin, curcumin, paclitaxel	pH/enzyme-responsive release; ↑ bioavailability (>30×); ↑ apoptosis; MDR reversal	Reduced side effects; enhanced therapeutic activity	Folic acid, RGD peptides, AS1411 aptamer, nucleolin targeting
Micelles	Amphiphilic block copolymers; hydrophobic core + hydrophilic shell; self-assembled	↑ solubility; drug protection; prolonged circulation; responsive to stimuli	Fisetin, Kushenol E, shikonin, ellagic acid	Protect from metabolism; ↑ Bax, caspases; inhibit PI3K/Akt, MAPK, VEGF; EMT suppression	Enhanced tumor inhibition; anti-angiogenesis; better survival; ↓ metastasis	RGD peptides; integrin targeting; pH/redox responsiveness
Nanocapsules	Drug-containing core with polymeric shell; well-defined structure; surface functionalizable	Drug protection; controlled release; high structural integrity	Cisplatin-NC combo, paclitaxel-lapatinib, hydrophobic NCs	Avoid efflux; ↑ intracellular delivery; co-target pathways; synergistic anticancer actions	90% tumor inhibition; overcome MDR; ↓ toxicity; better than free drugs	Folic acid, peptide ligands, antibodies; tumor microenvironment responsiveness
Nanostructured lipid carriers (NLCs)	Mixed solid-liquid lipid matrix (∼95 nm); second-gen nanocarriers	↑ drug loading and stability; zero-order release; oral delivery potential	Silibinin, cisplatin-NC combinations	Bypass efflux/P-gp; zero-order release; ↑ uptake; effective co-delivery	Overcome cisplatin resistance; improved patient adherence; synergistic tumor suppression	Folic acid, transferrin; receptor-mediated endocytosis

## 6 Extracellular vesicle-based delivery systems for NCs in OC therapy

Extracellular vesicles (EVs) are lipid bilayer-delimited particles secreted by most cell types and are significant for intercellular communication (Muttiah et al., 2024). EVs are primarily classified into three major types according to their size and biogenesis: exosomes, microvesicles, and apoptotic bodies (Jin et al., 2022). Exosomes are small EVs with diameters ranging from 30 to 150 nm and are generated through the endosomal pathway. They are generated as intraluminal vesicles within MVBs, from which they are secreted following the fusion of MVBs with the plasma membrane. Exosomes include proteins, including tetraspanins CD9, CD63, and CD81; lipids; and different species of RNA, which are derived from their parent cell and can function in signaling, particularly if exosomes are implicated in cancer or immune modulation (Han et al., 2022).

Microvesicles or ectosomes or shedding vesicles are 100–1,000 nm in diameter and are formed by direct outward budding of the plasma membrane. They are involved in various biological processes like angiogenesis, immune modulation, and metastasis, particularly in tumor microenvironments (Kumar M. A. et al., 2024). Apoptotic bodies are the largest EVs (one to five µm) and are derived from programmed cell death. These vesicles commonly carry cellular organelles and partially fragmented DNA and are implicated in cell corpse clearance and immune tolerance (Battistelli and Falcieri, 2020). Due to the size range overlap and because specific markers for this are not available, a simplified size-based nomenclature usually applies: small EVs (sEVs, <200 nm) and medium/large EVs (m/lEVs, >200 nm). Operational classification by ISEV has recommended an improved standardization and reproducibility of EVs research (Zhang et al., 2024; Ma B. et al., 2025).

**FIGURE 3:**
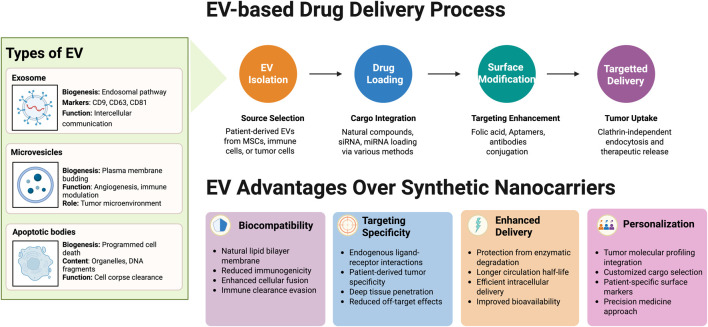
The extracellular vesicle-based delivery systems.

**FIGURE 4:**
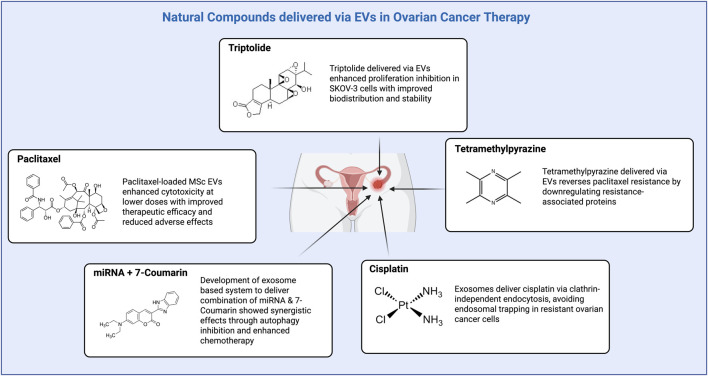
The NCs delivered via EVs in OC therapy.

Recent research indicates that secreted by nearly all types of cells that have emerged recently as highly promising nanocarriers for delivering drugs in cancer therapy (Fan et al., 2023; Barathan et al., 2024). From a biomedical perspective, exosome-mediated delivery possesses numerous advantages compared with conventional synthetic nanoparticles. These characteristics include enhanced protection of the therapeutic cargo from enzymatic degradation, longer circulation half-life, and deep penetration into tumor tissues. Their lipid bilayer membrane resembles endogenous cell membranes and thus can fuse with target cells and deliver intracellularly efficiently (Li J. et al., 2025). In comparison with synthetic nanocarriers, exosomes are a natural product of the body’s own cells and therefore have the potential to evade immune clearance and possess specific tumor targeting through endogenous ligand–receptor interactions (Chen et al., 2020). One of the most promising strategies involves the use of patient-derived exosomes as carriers. Exosomes from the patient’s own cells (mesenchymal stem cells, immune cells, or even tumor cells) can be loaded with therapeutic natural products, chemotherapeutic agents, or nucleic acids, and reimplanted into the patient (Lin et al., 2022). This autologous setup minimizes immunogenicity and enhances targeting, since such exosomes display surface markers and membrane proteins reflective of the patient’s own cellular environment, thus promoting tumor-specific uptake and reduced off-target effects. Although the majority of previous work is preclinical, early results suggest that exosomes hold promise to significantly improve the bioavailability and therapeutic window of natural products. For example, exosomes derived from MSCs and loaded with paclitaxel showed potent cytotoxicity in OC models at significantly lower doses, with improved therapeutic efficacy and diminished adverse effects compared to free paclitaxel (Yun et al., 2025). Meanwhile, another good example is triptolide, a highly anticancer diterpenoid triepoxide with no extensive clinical application due to toxicity and poor solubility. Triptolide encapsulated in exosomes (TP-EXOs) had enhanced inhibition of ovarian cancer cell proliferation (SKOV-3 cells) and suppressed tumor growth *in vivo* with improved biodistribution and stability compared to the free drug (Liu H. et al., 2019). Similarly, tetramethylpyrazine (TMP)-loaded exosomes have been demonstrated to reverse paclitaxel resistance in OC by downregulating resistance-associated proteins, resulting in significant tumor suppression (Zhao et al., 2024). Other natural molecules, such as β-elemene, have been encapsulated into exosomes to sensitize OC cells that are resistant to drugs via the downregulation of P-glycoprotein and enhancement of chemotherapy sensitivity (Liu W. et al., 2024). Another study demonstrated that exosomes could deliver cisplatin through clathrin-independent endocytosis, avoiding endosomal trapping and enhancing the drug’s anti-cancer effects in cisplatin-resistant ovarian cancer cells (Zhou et al., 2022). A novel exosome-based system was developed to deliver a combination of miRNA and 7-coumarin, achieving synergistic effects through autophagy inhibition and chemotherapy. This system improved drug targeting and efficacy against ovarian cancer cells (Hua et al., 2024). In addition, cargo customization based on tumor molecular profiling is an essential element of exosome therapy personalization. Next-generation sequencing and transcriptomic analysis of the patient’s tumor may unveil mutations, dysregulated signaling pathways (PI3K/Akt, JAK/STAT), and resistance-associated molecules (Lu W. et al., 2025). Exosomes can subsequently be designed to deliver payloads of interest such as siRNAs, miRNAs (eanti-miR-21-5p, miR-433 mimics), or plant molecules such as triptolide or curcumin, to target these molecular aberrations directly. This strategy ensures that the therapeutic payload disrupts tumor-driving mechanisms personalized for the patient (Ma et al., 2024). Further, surface modification of exosomes with targeting ligands also enhances specificity. For example, decoration of exosomes with folic acid, aptamers (AS1411), or antibodies against overexpressed ovarian cancer receptors (folate receptor α, integrin αvβ3) enhances their selective uptake and binding by tumor cells (He et al., 2022). These surface modifications enhance the therapeutic index and enable targeted therapy even in tumors with heterogeneous expression profiles. In spite of these promising findings, there are future challenges. These include the scalability and standardization of exosome isolation and purification, drug loading consistency, cargo release control, and long-term safety and biodistribution issues of engineered exosomes. It is necessary to overcome these challenges for the clinical translation of exosome-based systems (Dai and Shen, 2022). Figure 3 shows the concept of EVs as drug delivery system meanwhile Figure 4 explains the various example of EVs as drug delivery system in OC therapy with natural compounds.

## 7 Linking omics data to NCs in OC: emerging advances and future directions

Recent advances in omics technologies, such as genomics, transcriptomics, proteomics, metabolomics, and epigenomics, are transforming our understanding of OC biology at a very rapid rate (Subbannayya et al., 2021). In combination with NCs discovery, these technologies provide precious insights into molecular vulnerabilities, resistance mechanisms, and therapeutic targets. The single unifying theme of the research is the use of multi-omics integration to identify the essential molecular drivers of OC progression, stemness, and treatment response (Davis-Marcisak et al., 2021). For instance, single-cell RNA sequencing (scRNA-seq), CRISPR-based screens, and combined transcriptomic analysis have been employed to construct cancer stemness indices that can predict patient prognosis and the success of immunotherapies (Li Y. et al., 2025). Remarkably, genes such as CSE1L, which have been discovered by such platforms, are linked to poor prognosis and chemoresistance. This target information now presents specific molecular access points where natural compounds can be anticipated to have therapeutic effect (Auti et al., 2024). NCs such as polyphenols, flavonoids, alkaloids, and terpenoids are found to increasingly regulate key molecular pathways uncovered by omics platforms. These include traditional signaling pathways like NF-κB, PI3K/Akt, JAK/STAT, MAPK, and the pathways that control autophagy, apoptosis, and DNA repair from damage (Abdullah et al., 2025). Natural products, through their ability to interact with multiple nodes of these networks simultaneously, have the potential to surmount drug resistance and reduce metastasis, twin characteristics found by omics profiling uniformly in resistant ovarian tumors (Wolde et al., 2025).

Along with it, omics-driven biomarker discovery is also unlocking the doors to precision application of natural compounds. For instance, transcriptomic and metabolomic signatures can classify patients likely to be responsive to curcumin or resveratrol, which in turn gives rise to rationale combination therapy with chemotherapy or immune checkpoint agents (Wei et al., 2023). In this context, artificial intelligence (AI) and machine learning are stepping forward to integrate immense multi-omics data, enabling predictive modeling for natural compound activity based on every individual tumor molecular signature (Cui et al., 2025). Emerging multi-omics technologies are also revealing the molecular pathways by which natural products exert their anti-tumor effects. Metabolomics coupled with RNA sequencing has described how molecules like EGCG and berberine act on mitochondrial respiration, fatty acid metabolism, and immune cell recruitment to the tumor microenvironment. These are the secrets to rationalizing drug design and optimizing combination therapies (Sanadgol et al., 2025).

Future implications of this integrative strategy are vast. Customized drug delivery systems such as nanoparticle- or exosome-based carriers that could be designed based on patient-specific omics profiles to enhance delivery efficiency and reduce systemic toxicity (Chen et al., 2023). Liquid biopsy-derived exosomal omics data could enable dynamic tracking of drug response and real-time adjustment of therapeutic regimens with natural compounds (Boța et al., 2024). Additionally, systems biology approaches can facilitate the identification of synergistic combinations between natural compounds and current treatments, offering solutions to age-old challenges like platinum resistance (Wu J. et al., 2023).

## 8 Limitations and future directions

Although there is strong preclinical evidence for the multi-targeted anticancer potential of NCs in OC, translating them into the clinic remains a big challenge. Poor bioavailability and unfavorable pharmacokinetic properties, as in curcumin, resveratrol, and EGCG, result in low solubility, rapid metabolism, and systemic clearance, leading to sub-therapeutic concentrations at the tumor site and hence reduced *in vivo* efficacy (Trofin et al., 2025). Few large-scale, well-designed clinical trials have been performed, and most available studies have focused on the antioxidant effects of NCs against other cancers or in healthy populations rather than on OC-specific effects (Long et al., 2025). Few current clinical efforts are firmly based on sound preclinical rationale and investigate NCs as adjuncts to standard therapies rather than as active treatments per se. Further complicating this is the issue of dosing standardization, given the pronounced interpatient metabolic variability and heterogeneity between OC subtypes, making trial outcomes difficult to interpret (Liu B. et al., 2024). Important regulatory and perceptual hurdles persist, as NCs are often categorized as dietary supplements and therefore are generally excluded from demanding oncology development pipelines. In combination with their generally poor patentability and thus insufficient industrial investment, these factors hinder clinical translation and commercialization (Paller et al., 2016).

In summary, a number of strategic directions may help to overcome these limitations: Firstly, the development of advanced delivery systems such as polymeric nanoparticles, liposomes, dendrimers, NLCs, and biologically derived EVs should be one of the main focuses for improving the solubility, enhancing stability, controlling the release, and achieving tumor-targeted accumulation of NCs (Tripathi et al., 2024). These approaches will contribute to enhanced therapeutic efficacy while minimizing off-target toxicity and chemoresistance. The integration of multi-omics technologies, including genomics, transcriptomics, and proteomics, coupled with AI and machine learning, opens up a pathway toward precision medicine. This will allow for identifying predictive biomarkers for personalized NC therapy, helping to stratify OC patients most likely to benefit from specific NCs or combination regimens (Molla and Bitew, 2024). There is an urgent need for large, randomized controlled clinical trials focused specifically on OC to validate NCs not only as chemosensitizers but also as potential monotherapies in preventive or maintenance settings, using standardized high-quality formulations (Yan et al., 2020). Furthermore, synergy and combination strategies of NCs with conventional therapies, including platinum-based chemotherapy, PARP inhibitors, and immunotherapy, deserve further exploration (Molto Valiente and Amir, 2023). Elucidation of the mechanistic basis of such synergistic effects could enable the design of rational combination regimens that use lower doses of cytotoxic drugs with fewer side effects and overcome multidrug resistance (Duarte and Vale, 2022). Last but not least, sustainable sourcing and semi-synthesis of NCs are of critical importance in assuring reliable supply and ecological balance. Semi-synthetic derivatives and analogs with improved pharmacokinetic profiles and better patentability may facilitate pharmaceutical development and clinical acceptance (Musso and Biscussi, 2025). In summary, although the preclinical promise of NCs in OC therapy is great, an integrated approach that incorporates the newest drug delivery systems, omics-driven precision oncology, rational combination strategies, and sustainable production methods will be necessary to achieve their clinical potential. Well-funded, rigorously designed clinical trials to prove their efficacy and safety are a prerequisite for the translation of NCs into viable, evidence-based components of future ovarian cancer management.

## 9 Conclusion

NCs show multi-target anticancer activities against ovarian cancer by modulating various cellular pathways, crucial for the regulation of proliferation, apoptosis, angiogenesis, metastasis, and chemoresistance. Preclinically, compounds such as quercetin, curcumin, resveratrol, EGCG, berberine, ellagic acid, withaferin A, and celastrol have exhibited strong potential via different molecular modes of action, holding both therapeutic and chemosensitizing benefits. However, clinical application is still restricted due to issues of pharmacokinetic problems, non-standardized formulations, and inadequate OC-focused clinical trials. Improvement in nanotechnology-based delivery, omics-driven precision medicine, and combination strategies with existing chemotherapy and targeted agents may fill the gap in translation. Future studies should focus on thorough clinical validation, sustainable production, and regulatory recognition of NCs as valid therapeutic candidates. Overall, the integration of natural compounds into modern oncologic frameworks embodies a promising and sustainable strategy for improving the efficacy of ovarian cancer treatments while reducing therapeutic resistance.
